# Improving the Evidence on Long-Term Frailty Trajectories: Data from the Nurses’ Health Study

**DOI:** 10.1093/geroni/igaf122.4411

**Published:** 2025-12-31

**Authors:** Anna Siefkas, Ariela Orkaby, Marian Hannan, Sebastien Haneuse, A Heather Eliassen, Yuan Ma

**Affiliations:** Harvard. T.H. Chan School of Public Health, Boston, Massachusetts, United States; Brigham & Women’s Hospital, Harvard Medical School, Boston, Massachusetts, United States; Hebrew SeniorLife, Boston, Massachusetts, United States; Harvard. T.H. Chan School of Public Health, Boston, Massachusetts, United States; Harvard. T.H. Chan School of Public Health, Boston, Massachusetts, United States; Harvard. T.H. Chan School of Public Health, Boston, Massachusetts, United States

## Abstract

Evidence on long-term frailty trajectories is crucial for monitoring the dynamic changes in health status in older adults and may better inform prognosis than single-time measurements. However, such evidence is limited due to the scarcity of frailty data over decades as well as methodological challenges including missing data, attrition (study dropout), and truncation due to mortality. Leveraging decades-long data from the Nurses’ Health Study (1992–2016) and appropriate methods to address these challenges, we characterized long-term frailty trajectories of 89,312 women (aged 46–71 in 1992). A frailty index (FI) was calculated every four years, using two-stage multiple imputation with generalized linear mixed models to impute FI item non-response in years with available data (stage 1) and complete FI values in years of unit non-response (stage 2). We addressed attrition using inverse probability of censoring weighting. Models for frailty over time were estimated partly conditional on death and operationalized using GEE models with an independence working correlation structure to avoid implicit imputation of trajectories past the point of mortality. Mean FI values increased linearly by 0.0079 annually in the population (95% CI: 0.0078 – 0.0080), adjusting for age. Trajectories differed by baseline frailty status and by age, however, greater variation in trajectories was seen by frailty status than by age. The group that was prefrail at baseline demonstrated the most rapid increase in FI over time. Our results build upon prior findings of increasing FI values over time and provide a framework for rigorous longitudinal studies of aging.

